# Neoadjuvant chemotherapy prior to radical cystectomy for non-muscle invasive bladder cancer: national trends and pathologic outcomes

**DOI:** 10.3389/fonc.2024.1392062

**Published:** 2024-05-13

**Authors:** Kailey Davis, Jeffrey Orf, Eric Ballon-Landa, Zachary Hamilton

**Affiliations:** Division of Urology, Department of Surgery, Saint Louis University, St. Louis, MO, United States

**Keywords:** neoadjuvant chemotherapy, radical cystectomy (RC), Non-muscle invasive bladder cancer (NMIBC), clinical practice patterns, complications

## Abstract

**Background:**

There is a sparsity of literature on treatment outcomes for patients with non-muscle invasive bladder cancer (NMIBC) who received neoadjuvant chemotherapy (NAC). We aim to analyze the outcomes associated with the use of NAC prior to radical cystectomy for NMIBC utilizing the National Cancer Database.

**Materials/Methods:**

The National Cancer Database bladder dataset was evaluated for patients with NMIBC and known pT staging undergoing RC from 2006–2016. The primary outcome was the utilization of NAC. The secondary outcomes were pathologic down staging to pT0, positive surgical margins, 30-day readmission, and overall survival.

**Results:**

The proportion of patients receiving NAC prior to radical cystectomy for NMIBC increased from 8.6% in 2006 to 14.8% in 2016. Those who received NAC had significantly higher tumor stages (cT1 vs cTa/is) with 85.7% of patients receiving NAC presenting with cT1 as opposed to only 82% in those not receiving NAC (p < 0.001). Similarly, there were significantly more patients who were cN+ in the NAC group as compared to those who did not receive NAC (5.5% vs. 1.1%, p < 0.001). For patients who received NAC, the rate of downstaging to pT0 was 12.7% as compared to only 3.3% in patients who did not receive NAC (p < 0.001). There was no significant difference comparing the rates of positive margins or 30-day readmissions between groups. On multivariable logistic regression for pathologic downstaging, NAC was significant (OR 4.1, p < 0.05). There was no significant difference in overall survival between patients treated with or without NAC.

**Conclusion:**

NAC prior to RC in patients with NMIBC has increased in recent years and correlates with tumor downstaging. Further research is requisite to identify patients who obtain the greatest benefit of NAC in the NMIBC setting.

## Introduction

As the 6th most common cancer in males and 10th most common cancer regardless of sex, bladder cancer affects nearly 575,000 people worldwide based on 2020 data (1). Seventy-five percent of these patients present with non-muscle invasive bladder cancer (NMIBC). With such a significant proportion of bladder cancer patients being diagnosed with NMIBC, it is important to optimize treatment and decrease the risk of progression to muscle invasive disease.

The current treatment paradigm for non-muscle invasive bladder cancer is broad, ranging from more conservative options, such as endoscopic management with intravesical therapy, to early radical cystectomy. The use of neoadjuvant chemotherapy (NAC) prior to radical cystectomy in patients with muscle invasive disease is the standard of care, which is associated with pathological downstaging and improved overall survival (2). Recommendation for radical cystectomy (RC) for patients with NMIBC has been included in both the European Association of Urology (EAU) and American Urological Association (AUA) guidelines, specifically for patients with recurrent or aggressive disease, but these guidelines do not make recommendations for NAC in the NMIBC setting (3, 4). However, there is a sparsity of literature on treatment outcomes for patients with non-muscle invasive bladder cancer who received NAC. Previous literature has found that up to 50% of resected NMIBC are upstaged to muscle-invasive tumors after cystectomy, thus it stands to reason that a proportion of patients with NMIBC could gain the benefits of NAC prior to radical cystectomy (5).

The primary goal of this study was to evaluate the trends in use of NAC prior to RC in patients with NMIBC. Our secondary goal was to determine the effects on tumor characteristics and patient outcomes. We hypothesized that use of NAC would increase over time and would contribute to better pathologic and patient outcomes, thus representing an advancement in the treatment of NMIBC.

## Materials and methods

The NCDB is a joint quality improvement project produced by the American Cancer Society and the American College of Surgeons, which captures data from 30% of United States hospitals and approximately 70% of all patients newly diagnosed with cancer (6). The data collected is de-identified and the categories include patient socio-demographics, tumor characteristics, clinical and pathologic staging, definitive treatments, and all-cause mortality. Details of specific chemotherapy regimens and cancer-specific survival are not included. All information is collected in a HIPAA compliant manner. Data submitted to the NCDB undergoes extensive quality monitoring and validity reviews on an annual basis. The data utilized in this study came from the publicly shared and de-identified NCDB data set. Approval by the institutional review board was not necessary because no patient or hospital identifiers were analyzed. The American College of Surgeons and the Commission on Cancer have not verified and are not responsible for analytic or statistical methodology employed or the conclusions drawn from these data by the investigator.

The NCDB bladder cancer dataset was queried for patients who were diagnosed with cT1, cTis, or cTa disease from 2006 to 2016 and underwent subsequent RC with or without NAC. Patients with cM positive disease were excluded and all cN stages were included. All patients undergoing RC were stratified by receipt of NAC for purposes of analysis. Clinical information analyzed included age, gender, race, Charlson score, facility type, income status, insurance status, tumor grade, presence of variant histology, and clinical stage. Of note, treatment facility type was categorized as low volume or high volume. Treatment facilities that accrued 500 or more newly diagnosed cancer cases per year were considered high-volume (including academic centers and comprehensive cancer centers), whereas facilities with less than 500 were labeled low-volume (including community and integrated network cancer programs), as per NCDB stratification. Perioperative and survival parameters analyzed included length of hospital stay, final pathologic stage, positive margins, robotic approach, unplanned 30-day readmission after surgery, length of follow up, and mortality (overall, 30-, and 90-day).

Student’s T-test was performed for continuous variables, and Fischer’s exact or Pearson chi-square tests for categorical variables. Analysis was performed comparing NAC cohorts, and an additional analysis was performed stratified by pT0 status. Linear regression analysis was used to determine the rate of NAC use over time. Logistic regression analysis for NAC use was determined by variables that were statistically significant on univariate analysis and included age, race, Charlson score, clinical stage (including cT and cN stage), and income status. An additional logistic regression for pathologic down staging to pT0 disease included age, clinical stage (cT and cN), urothelial pathology, and NAC use. Lastly, Kaplan-Meier analysis for overall survival was performed between NAC cohorts. We utilized SPSS v27 (New York, United States) for all analyses, with p value of <0.05 denoting statistical significance. Our primary outcome was NAC utilization over time. Our secondary outcomes included predictors of NAC receipt and overall survival.

## Results

**Figure 1:**
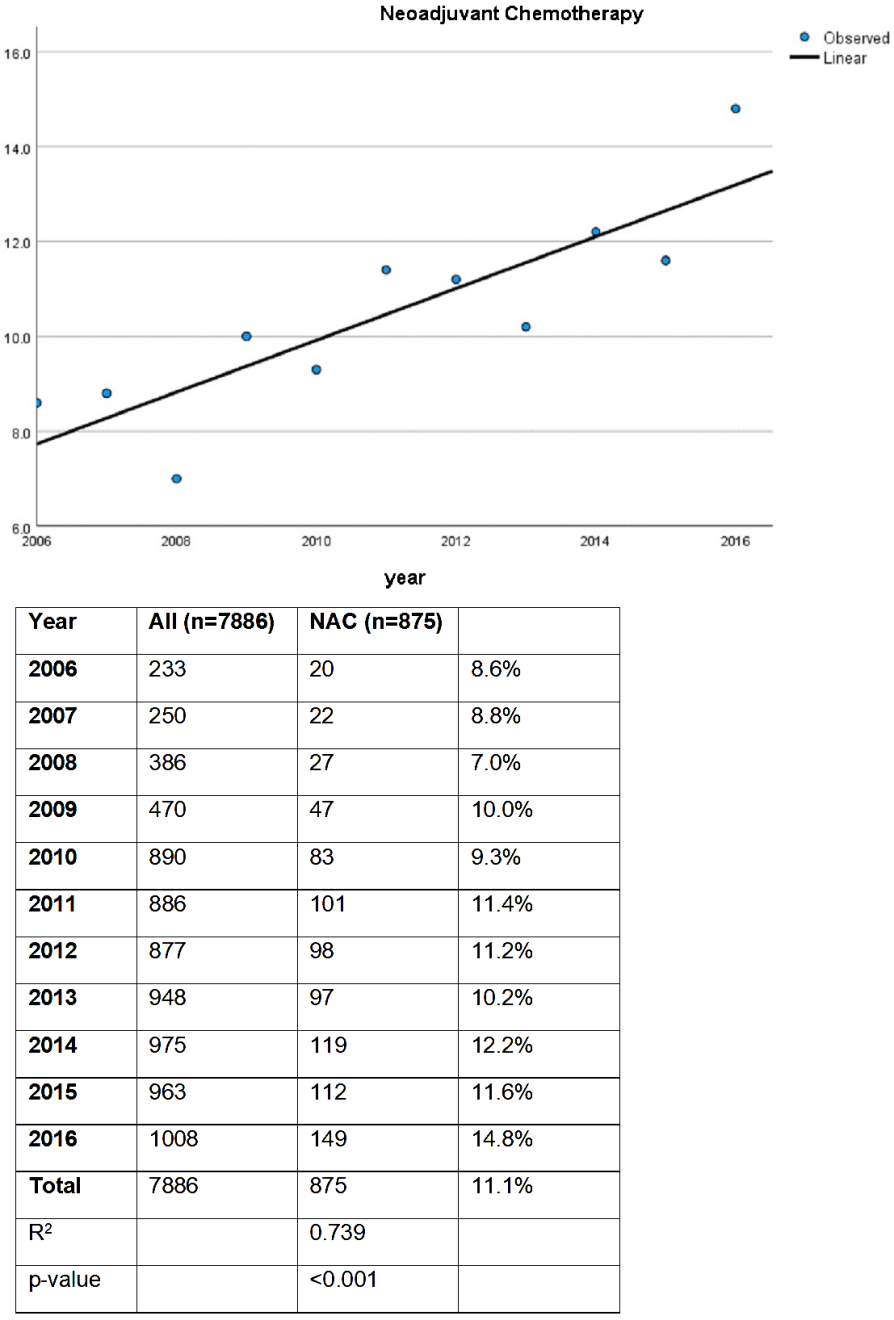
NAC Use Over Time.

In this study, a total of 7886 patients from the NCDB who underwent radical cystectomy for NMIBC were included and stratified by receipt of NAC. For the entire study period, 11% of patients received NAC. On linear regression analysis, there was a statistically significant increase in use of NAC prior to RC from 2006 to 2016 (**Figure 1**, p < 0.001). In 2006, 8.6% received NAC prior to RC, while 14.6% received NAC prior to RC in 2016 (R^2^ 0.739).

Demographics and clinical tumor characteristics are outlined in **Table 1**. Patients receiving NAC were significantly younger (66.0 ± 10.0 vs 67.4 ± 10.5 years, p < 0.001); however, race and sex were similar between cohorts. NAC patients had higher income status (p = 0.05) and significant lower Charlson Comorbidity Index (CCI, p = 0.047). For clinical tumor stage, patients with NAC had higher rates of cT1 and cTis but a lower rate of cTa disease, compared to non-NAC (p < 0.001). The type of treatment facility did not differ between NAC cohorts. For example, patients without NAC had cT1 cancer 81.9% of the time compared to 85.7% of patients with NAC. Furthermore, the rate of clinically node-positive cancer prior to cystectomy (cN+) was significantly higher in NAC patients (5.5% vs 1.1%, p < 0.001). No differences were noted in variant pathology or nuclear grade between NAC and non-NAC groups.

**Table 1:** Patient demographics and clinical tumor characteristics.

Variable	All (n=7886)	No NAC (n=7011)	NAC (n=875)	p-value
**Mean Age**	67.2 ± 10.4	67.4 ± 10.5	66.0 ± 10.0	<0.001
Race				0.051
**White**	7110 (90.2%)	6307 (90.0%)	803 (91.8%)	
**Black**	479 (6.1%)	442 (6.3%)	37 (4.2%)	
**Other**	297 (3.8%)	262 (3.7%)	35 (4.0%)	
**Male**	6151 (78.0%)	5482 (78.2%)	669 (76.5%)	0.243
Income Status				0.050
**<$38,000**	848 (11.1%)	777 (11.4%)	71 (8.4%)	
**$38,000–47,999**	1303 (17.0%)	1158 (17.0%)	145 (17.1%)	
**$48,000–62,999**	2117 (27.7%)	1884 (27.7%)	233 (27.5%)	
**$63,000+**	3382 (44.2%)	2985 (43.9%)	397 (46.9%)	
**Uninsured**	179 (2.3%)	165 (2.4%)	14 (1.6%)	0.185
**High Volume Facility**	6587 (83.5%)	5848 (83.4%)	739 (84.5%)	0.468
Charlson				0.047
**0**	5400 (68.5%)	4766 (68.0%)	634 (72.5%)	
**1**	1784 (22.6%)	1617 (23.1%)	167 (19.1%)	
**2**	516 (6.5%)	461 (6.6%)	55 (6.3%)	
**3+**	186 (2.4%)	167 (2.4%)	19 (2.2%)	
cT Stage				<0.001
**cTa**	883 (11.2%)	822 (11.7%)	61 (7.0%)	
**cTis**	514 (6.5%)	450 (6.4%)	64 (7.3%)	
**cT1**	6489 (82.3%)	5739 (81.9%)	750 (85.7%)	
**cN+**	125 (1.6%)	77 (1.1%)	48 (5.5%)	<0.001
Pathology				0.522
**Urothelial**	7048 (89.4%)	6260 (89.3%)	788 (90.1%)	
**Variant**	838 (10.6%)	751 (10.7%)	87 (9.9%)	
**High Nuclear Grade**	6008 (76.2%)	5334 (76.1%)	674 (77.0%)	0.556

In **Table 2**, we examined both perioperative and survival outcomes. No differences in length of stay, 30-day readmission rates, or duration of follow-up were noted between NAC and non-NAC groups. Rate of positive margins after cystectomy and overall survival were also similar. Rates of pathologic downstaging to pT0 were higher in the NAC cohort (12.7% vs 3.4%, p < 0.001). Additionally, the rate of pathologic node positive disease (pN+) was higher in the NAC cohort (16.0% vs 12.8%, p = 0.009). NAC patients had improved 30-day mortality outcomes (0.9% vs 1.9%, p = 0.042) but no differences in 90-day mortality or overall survival.

**Table 2:** Perioperative and survival outcomes.

Variable	All (n=7886)	No NAC (n=7011)	NAC (n=875)	p-value
**Length of Stay**	8.9 ± 9.0	9.0 ± 9.1	8.5 ± 8.0	0.116
**30 day Readmit**	686 (8.7%)	611 (8.7%)	75 (8.6%)	0.949
pT Stage				<0.001
**pT0**	348 (4.4%)	237 (3.4%)	111 (12.7%)	
**pT1/a/is**	4343 (55.1%)	3928 (56.0%)	415 (47.4%)	
**pT2**	1375 (17.4%)	1215 (17.3%)	160 (18.3%)	
**pT3**	1222 (15.5%)	1103 (15.7%)	119 (13.6%)	
**pT4**	598 (7.6%)	528 (7.5%)	70 (8.0%)	
**pN+**	1034 (13.1%)	894 (12.8%)	140 (16.0%)	0.009
**Positive Margin**	616 (7.8%)	557 (7.9%)	59 (6.7%)	0.229
**Robotic Approach**	1454 (18.4%)	1264 (18.0%)	190 (21.7%)	0.010
**Length of Follow Up (months)**	40.5 ± 29.1	40.6 ± 29.2	39.9 ± 28.3	0.548
**Mortality (all pts)**	2565 (32.5%)	2306 (32.9%)	259 (29.6%)	0.051
**Within 30 Days of Treatment**	142 (1.8%)	134 (1.9%)	8 (0.9%)	0.042
**Within 90 Days of Treatment**	331 (4.2%)	304 (4.3%)	27 (3.1%)	0.089

Using preoperative variables, we performed a logistic regression analysis to identify predictors of receiving NAC, included in **Table 3**. Factors significantly associated with increased odds of NAC included cT stage (OR 1.731 and 1.951 for cT1 and cTis, respectively), cN+ status (OR 4.948), and yearly income over $63,000 (OR 1.397).

**Table 3:** Logistic regression for NAC.

Variable	OR	95% CI low	95% CI high	p-value
**Age**	.989	.982	.996	.**002**
Race (white ref)				
Black	.708	.497	1.008	.056
Other	1.028	.710	1.487	.884
Income Status (<$38,000 ref)				
$38,000–47,999	1.328	.981	1.798	.066
$48,000–62,999	1.317	.992	1.750	.057
**$63,000+**	1.397	1.065	1.833	.**016**
Charlson Score (3+ ref)				
0	1.084	.665	1.768	.747
1	.854	.514	1.420	.543
2	1.036	.593	1.812	.901
cT Stage (cTa ref)				
**cT1**	1.731	1.307	2.292	<.**001**
**cTis**	1.951	1.335	2.850	<.**001**
**cN+**	4.948	3.387	7.227	<.**001**

Using post-resection findings, **Table 4** compares demographic and clinical tumor characteristics between patients who were downstaged to pT0 and all other stages (pT1-pT4). Notably, mean patient age, cT stage prior to cystectomy, cN+ status, and receipt NAC prior to RC were significantly different between cohorts. When comparing the use of NAC prior to RC, 31.9% of patients with pT0 downstaging received NAC compared to 10.1% of patients with residual pT1-pT4 disease (p < 0.001). Race, sex, Charlson Comorbidity Index, income status, high-volume facility, insurance status, and presence of high nuclear grade tumors were not statistically significant between groups. **Table 5** uses logistic regression analysis to identify factors associated with pT0 downstaging was performed, including age, cT stage, cN stage, histology, and NAC use. Treatment with NAC was significantly associated with a 4x higher rate of downstaging (OR 4.045, p < 0.001).

**Table 4:** Patient demographics and clinical tumor characteristics.

Variable	All (n=7886)	All other stages (n=7538)	pT0 (n=348)	p-value
**Mean Age**	67.2 ± 10.4	67.3 ± 10.4	64.8 ± 9.9	<0.001
Race				0.966
**White**	7110 (90.2%)	6795 (90.1%)	315 (90.5%)	
**Black**	479 (6.1%)	459 (6.1%)	20 (5.7%)	
**Other**	297 (3.8%)	284 (3.8%)	13 (3.7%)	
**Male**	6151 (78.0%)	5876 (78.0%)	275 (79.0%)	0.691
Income Status				0.456
**<$38,000**	848 (11.1%)	818 (11.2%)	30 (8.9%)	
**$38,000–47,999**	1303 (17.0%)	1244 (17.0%)	59 (17.5%)	
**$48,000–62,999**	2117 (27.7%)	208 (27.7%)	89 (26.3%)	
**$63,000+**	3382 (44.2%)	3222 (44.1%)	160 (47.3%)	
**Uninsured**	179 (2.3%)	169 (2.2%)	10 (2.9%)	0.458
**High Volume Facility**	6587 (83.5%)	6287 (83.4%)	300 (86.2%)	0.183
Charlson				0.570
**0**	5400 (68.5%)	5154 (68.4%)	246 (70.7%)	
**1**	1784 (22.6%)	1710 (22.7%)	74 (21.3%)	
**2**	516 (6.5%)	498 (6.6%)	18 (5.2%)	
**3+**	186 (2.4%)	176 (2.3%)	10 (2.9%)	
cT Stage				<0.001
**cTa**	883 (11.2%)	865 (11.5%)	18 (5.2%)	
**cTis**	514 (6.5%)	505 (6.7%)	9 (2.6%)	
**cT1**	6489 (82.3%)	6168 (81.8%)	321 (92.2%)	
**cN+**	125 (1.6%)	114 (1.5%)	11 (3.2%)	0.025
Pathology				<0.001
**Urothelial**	7048 (89.4%)	6767 (89.8%)	281 (80.7%)	
**Variant**	838 (10.6%)	771 (10.2%)	67 (19.3%)	
**High Nuclear Grade**	6008 (76.2%)	5755 (76.3%)	253 (72.7%)	0.122
**Neoadjuvant Chemo**	875 (11.1%)	764 (10.1%)	111 (31.9%)	<0.001

**Table 5:** Logistic regression for pT0.

Variable	OR	95%CI low	95%CI high	p-value
**Age**	.980	.970	.990	<.**001**
cT Stage (cTa ref)				
**cT1**	2.126	1.308	3.453	.**002**
**cTis**	.746	.331	1.682	.480
cN+	1.121	.584	2.150	.732
**Urothelial**	.498	.375	.661	<.**001**
**NAC**	4.045	3.174	5.154	<.**001**

**Figure 2:**
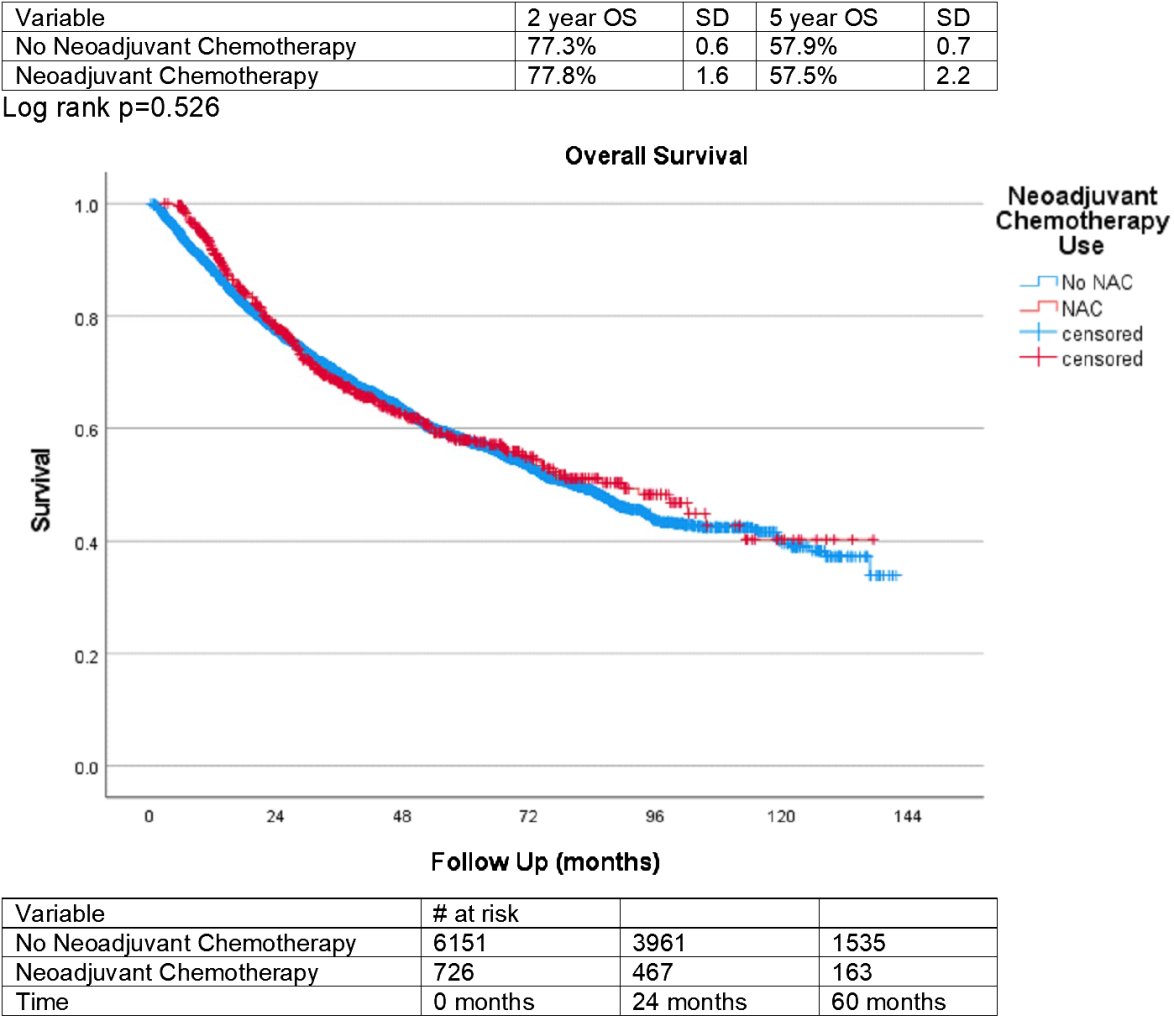
Kaplan Meier Overall Survival. Log rank p=0.526.

Finally, overall survival for those treated with NAC and those who did not receive NAC was plotted on a Kaplan-Meyer curve in **Figure 2**. This was statistically insignificant between groups, with a p-value of 0.526. Overall survival after 5 years was 57.9% (SD = 0.7) for patients not treated with NAC and 57.5% (SD = 2.2) for those treated with NAC.

## Discussion

In this study, we present a review of data spanning 10 years that examines the use of NAC prior to RC for patients with NMIBC. Notably, patients were more likely to receive NAC prior to RC if they had a higher income status, a Charlson Comorbidity Index of 0, cTis/cT1 tumors, or clinically node-positive cancer; however, treatment facility did not correlate with receipt of NAC. Additionally, after RC was completed, patients treated with NAC prior to surgery were more likely to have pathologic downstaging to pT0 than their non-NAC counterparts. NAC has been steadily increasing in the years since data collection began, even though it is not a standardized treatment for NMIBC in AUA or EUA guidelines. The increase in use of NAC prior to RC over time may show an increase in clinicians’ comfort and familiarity with this treatment modality.

When examining trends across patient demographics, higher rates of NAC prior to RC is seen for patients with higher income status, and this finding coincides with known socio-economic disparities in bladder cancer. This has previously been identified in the muscle invasive setting for bladder cancer, as well (7). Lower comorbidity status and higher risk clinical staging with cTis/cT1 disease are associated with increased use of NAC. We hypothesize that patients with higher preoperative tumor risk factors may be provided NAC due to concern for pre-cystectomy understaging and clinicians may favor the use of NAC in patients with lower comorbidity due to improvements in tolerance of chemotherapy regimens. Furthermore, patients with clinically node-positive cancer prior to cystectomy are likely to have the most benefit from systemic chemotherapy due to the risks of metastatic disease without treatment. This correlates with our findings of increased use of NAC among patients with node-positive cancer. Additional high-risk tumor factors such as size, multifocality, presence of invasion into surrounding tissues or vascular structures, and mass effect on nearby structures (i.e. hydronephrosis) are not available with the queried dataset, however such knowledge would provide additional insight into trends among patients treated with NAC prior to RC.

A very interesting and encouraging outcome of our analysis was the rate of pathologic downstaging to pT0 among those who were treated with NAC prior to RC. The rate of pT0 at cystectomy was four times higher with NAC on multivariate analysis as well. This finding suggests there are patients with NMIBC who may derive oncologic benefits from NAC and may encourage providers to utilize NAC prior to RC in certain clinical scenarios. This improvement with downstaging is also seen in the muscle invasive setting and associated with long-term improvement in survival (8). It may be that the patients in this analysis who received pT0 downstaging had underlying muscle invasive disease that was not diagnosed prior to radical cystectomy. It is also possible that these patients had specific histopathology that was chemo-sensitive. The underlying drivers of downstaging are unknown based on this dataset but our analysis serves to generate hypotheses and encourage further research.

The survival outcomes of patients in our study were statistically insignificant, based on NAC utilization. At both two and five years of follow-up, the overall survival is very similar between NAC and non-NAC groups. This finding indicates that NAC prior to RC may not contribute to overall survival in the NMIBC setting, which contrasts with the known beneficial effect of NAC on muscle invasive disease (2). One potential driver of this finding is a relatively slower progression of NMIBC compared to more aggressive muscle invasive disease. Furthermore, the specific chemotherapy regimens are not detailed in the NCDB so we cannot ensure that all patients received platinum-based chemotherapy with standardized regimens. However, based on the other findings of the study, treatment with NAC likely confers the clinical benefit of downstaging NMIBC tumors prior to radical cystectomy.

At this time, AUA and EAU guidelines do not provide directed recommendations for the use of NAC prior to radical cystectomy for patients with NMIBC (3, 4). It is possible that the lack of survival benefit for patients treated with NAC, as detailed above, is the main contributor to the exclusion of NAC in the current treatment paradigm. However, considering the lack of available data on systemic neoadjuvant chemotherapy prior to radical cystectomy, our analysis may provide the impetus for opening a new avenue of investigation into NAC prior to radical cystectomy as an available treatment pathway for certain patients.

This study illuminates important trends in the possibilities for treatment of NMIBC, however there are limitations that must be acknowledged. Limitations of the study include inherent selection bias due to this being a retrospective study. Considering the nature of aggregate data, we are unable to determine certain specifics about patient treatment, such as clinical decision-making algorithms for the use of NAC in the setting of NMIBC. Information that may be helpful for future studies include individual treatment regimens or prior interventions performed before definitive treatment with RC, such as intravesical chemotherapy or use of BCG. Lastly, this data cannot be followed to determine progression or downstaging of individual tumors and data on recurrence rates are not available within the NCDB.

## Conclusion

NAC prior to radical cystectomy in patients with NMIBC has increased in recent years and is associated with tumor downstaging but did not improve overall survival. Further research is requisite to identify patients who obtain the greatest benefit of NAC in the NMIBC setting.

## Data availability statement

The raw data supporting the conclusions of this article will be made available by the authors, without undue reservation.

## Ethics statement

The requirement of ethical approval was waived by Saint Louis University, Institutional Review Board for the studies involving humans because National dataset, all patient information de-identified. The studies were conducted in accordance with the local legislation and institutional requirements. Written informed consent for participation was not required from the participants or the participants’ legal guardians/next of kin because National dataset, retrospective, all patient information de-identified, formal consent not possible.

## Author contributions

KD: Writing – review & editing, Writing – original draft. JO: Writing – review & editing, Investigation, Conceptualization. EB: Writing – review & editing, Methodology, Investigation, Formal analysis. ZH: Writing – review & editing, Writing – original draft, Project administration, Methodology, Investigation, Formal analysis, Conceptualization.
